# Lymphotoxins Serve as a Novel Orchestrator in T1D Pathogenesis

**DOI:** 10.3389/fimmu.2022.917577

**Published:** 2022-06-09

**Authors:** Shi-Wei Liu, Fei Sun, Shan-Jie Rong, Ting Wang, Cong-Yi Wang

**Affiliations:** ^1^ Department of Endocrinology, Shanxi Bethune Hospital, Shanxi Academy of Medical Sciences, Tongji Shanxi Hospital, Third Hospital of Shanxi Medical University, Taiyuan, China; ^2^ Department of Respiratory and Critical Care Medicine, The Center for Biomedical Research, National Health Commission (NHC) Key Laboratory of Respiratory Diseases, Tongji Hospital, Tongji Medical College, Huazhong University of Sciences and Technology, Wuhan, China

**Keywords:** type 1 diabetes (T1D), lymphotoxins, tertiary lymphoid organ (TLO), immune regulation, insulitis

## Abstract

Type 1 diabetes (T1D) stems from pancreatic β cell destruction by islet reactive immune cells. Similar as other autoimmune disorders, there is no curative remedy for T1D thus far. Chronic insulitis is the hallmark of T1D, which creates a local inflammatory microenvironment that impairs β cell function and ultimately leads to β cell death. Immune regulation shows promise in T1D treatment by providing a time window for β cell recovery. However, due to the complex nature of T1D pathogenesis, the therapeutic effect of immune regulation is often short-lasting and unsatisfying in monotherapies. Lymphotoxins (LTs) were first identified in 1960s as the lymphocyte-producing cytokine that can kill other cell types. As a biological cousin of tumor necrosis factor alpha (TNFα), LTs play unique roles in T1D development. Herein in this review, we summarized the advancements of LTs in T1D pathogenesis. We particularly highlighted their effect on the formation of peri-islet tertiary lymphoid organs (TLOs), and discussed their synergistic effect with other cytokines on β cell toxicity and autoimmune progression. Given the complex and dynamic crosstalk between immune cells and β cells in T1D setting, blockade of lymphotoxin signaling applied to the existing therapies could be an efficient approach to delay or even reverse the established T1D.

## Introduction

Type 1 diabetes (T1D) is a prototypical autoimmune disease featured by islet β cell destruction along with absolute insulin deficiency and uncontrolled autoreactive immune attack (1). Genetic factors contribute to T1D susceptibility and play a pivotal part in disease initiation and progression. In general, the polymorphisms conferring T1D risk predominantly locate within loci that are either associated with β cell functionality (e.g., *Mda5*, *Ptpn2*, *Usp18* and *Glis3*) or linked to autoimmune responses (e.g., *HLA, IL-2, IL-2rα, Ctla4* and *Stat3*) (2, 3). These findings laid a foundation for the existence of a vicious crosstalk between immune cells and β cells, which forms a positive feedback loop hard to be halted by the existing therapeutics in clinical settings (4). Moreover, environmental perturbations (such as viral infections, exposure of toxic pollutants, microbiota alteration, diet, exercise and lifestyle changes) linked to epigenetic machineries (e.g., DNA methylation, histone or protein post-translational modifications, and non-coding RNAs) modulate the initiation, progression and remission process of T1D (5, 6). Altogether, these etiological cues make T1D a chronic and complex disease.

The immune factors are crucial in mediating the crosstalk between immune cells and β cells. Particularly, inflammatory cytokines produced by dendritic cells (DCs), macrophages, natural killer (NK) cells, T cells and B cells could act on β cells and impair β cell function by eliciting endoplasmic reticulum (ER) stress, oxidative stress and mitochondrial dysfunction (7). CD4^+^ helper T cells (Th) and CD8^+^ cytotoxic T cells (CTLs) are the major subsets responsible for islet destruction (8). Effector Th cells (Teffs) directly secrete IFN-γ, IL-1β and other soluble mediators such as nitric oxide (NO) to induce β cell death (9), or indirectly motivate CTLs to release granzymes and perforin to exert the killing effect (10). Teffs contain several distinct subpopulations, among which Th1 and Th17 play the most critical role. Th1 cells are the well-recognized β cell destroyers due to their capacity of producing massive type 1 cytokines (e.g., IFN-γ, TNF-α, IL-12 and so on) (11). In contrast, the pathogenicity of Th17 is controversial (12, 13). It is generally accepted that Th17 cells polarized by IL-6, IL-23 and IL-1β *in vitro* are pathogenic (termed as pTh17), and diabetogenic Th17 cells display characteristics resembling those of Th1 cells (11, 12). Therefore, the Th1 derived cytokines, IL-1β, IFN-γ and TNFα, constitute the classical trio frequently used to induce β cell dysfunction *in vitro* (14). Likewise, stressed or damaged β cells actively secret cytokines and chemokines to propagate the self-attacking program. Notably, β cells serve as the main source of CXCL10, and the highest CXCL10 expression has been noted at the time of diagnosis rather than the chronic phase of T1D development (15, 16). Concurrently, CXCR3, the receptor for CXCL10, is detected on islet-infiltrating lymphocytes of recent-onset T1D patients. Blockade of the CXCL10/CXCR3 axis with neutralizing antibody reduced T1D incidence in mice, and hopefully, a persistent remission of T1D could be achieved once anti-CD3 therapy is combined (17). Collectively, these studies highlight the complex interaction between immune cells and β cells, which alternatively explain why monotherapies targeting on a single molecule or signaling pathway are unsatisfying in T1D treatment.

Tumor necrosis factor superfamily members (TNFSF), through binding to their corresponding receptors (TNFRSF), affect both immune system and function of β cells. The TNFSF/TNFRSF axes regulate basic biological processes such as cell survival, differentiation, activation, and immune cell development by utilizing different sets of downstream adaptors (18, 19). Specifically, TNFRSF with cytoplasmic death domain (DD) recruits TRADD and FADD, initiating apoptotic pathway *via* the activation of Caspases (20). TNFRSF with non-death domain in the cytoplasmic tail recruits TRAF 1-6 (21), which in turn activates serine/threonine kinases such as MAPK, AKT and NF-κB, and induces the expression of cyclins and inflammatory cytokines. There are over 20 TNFSF/TNFRSF pairs in the superfamily, and many of them are critically engaged with T1D pathogenesis. Based on the action mode, they can be classified into two groups. One group contains members capable of inducing β cell apoptosis through FasL/Fas, TNFα/TNFR1 (13, 22) and TRAIL/TRAILR, while the other group function through immune modulation, which includes OX40L/OX40, CD40L/CD40, CD70/CD27, 4-1BBL/4-1BB, RANKL/RANK, BAFF/BAFFR, GITRL/GITR etc. (23). However, this simplified dichotomy does not mean each TNFSF/TNFRSF pair has only confined regulatory function. For instance, TNFR1 expressing on β cells is related to the initiation of apoptotic pathway, while its expression in regulatory T (Treg) cells affects Treg suppressive capacity (24, 25). Lymphotoxins (LTs) belong to the TNFSF superfamily and are potent immune factors to mediate the crosstalk between immune cells and β cells. Herein, we intend to summarize the advancement of lymphotoxins in T1D pathogenesis. Discussion would be focused on the related mechanisms in the formation of tertiary lymphoid organs (TLOs), induction of β cell dysfunction and enhancement of autoimmune responses during T1D development.

## LTs and Their Cognate Receptors in T1D Pathogenesis

LTs share approximately 35% sequence homology with TNFα, and they are evolutionarily correlated and employ the same receptors (TNFR1 and TNFR2) (26). However, LTs differ from TNFα in many aspects, including the source of producing cell types, the working mechanism, and the resulting biological consequences. LTs, consisting of LTA (TNFSF1, also known as TNFβ) and LTB (TNFSF3), are produced by lymphoid tissue inducer (LTi), Th1, CD8 T cells, NK cells, B cells, and innate lymphoid cells (ILCs) (27–29). LTs exist in the form of LTα3, which is a soluble homotrimer that binds to TNFR1, TNFR2, HVEM (herpes virus entry mediator, minor binding) and signals through the canonical NF-κB pathway (30); or in the form of LTα1β2, which is a membrane-bound heterotrimer that binds to LTβR and activates alternative NF-κB pathway (31). The LT receptors display distinct expression patterns and a division of labor. TNFR1 and TNFR2 are present on most cell types, mediating the processes of apoptosis, inflammation and immune activation, while LTβR is predominantly expressed on monocytes, DCs, follicular DCs (FDCs), endothelial cells, epithelial cells and lymphoid stromal cells, contributing to the ectopic formation of TLOs (32). These unique features of LT-LT receptor signaling render LTs a pleiotropic player in the process of infection, autoimmunity, development of tumor and many other immune related disorders.

The LT genes situate in the MHC cluster, mapped between class III and class I genes and near the TNFα locus. From the perspective of evolution, LTA, LTB and TNFα probably arise from the process of gene duplication. Genetic studies suggest that the TNFα polymorphisms (308 A/G and 857 T/C in particular) confer T1D risk (33). Although the LTA genetic polymorphism has been linked to T1D (34), the supporting evidence is unsolid (35). Moreover, blockade of LT-LT receptor signaling has been proven effective to prevent T1D in mice. Studies in nonobese diabetic (NOD) mice (a mouse model of human T1D) with TNFR1 deficiency abrogated T1D progression (36). Adoptive transfer of either splenocytes or TCR transgenic CD8 T cells demonstrated the existence of two distinct diabetogenic pathways: one is TNFR1 independent which is predominantly caused by the cytotoxic effect of CD8 T cell; the other is TNFR1 dependent which relies on the impact of TNFα and LT (36). In particular, pregnant NOD mice treated with LT-beta receptor immunoglobulin fusion protein (LTbetaR-Ig) on embryonic day 11 (E11) and E14 generated offspring absent of insulitis and free of diabetes at 12-month of age (37). Similarly, embryonic expression of LTbetaR-Ig blocked the development of diabetes in NOD mice. However, the protective effect wanes once the concentration of chimeric LTbetaR-Ig declines (38). Taken together, these studies demonstrated convincing evidence supporting the implication of LTs in T1D pathogenesis.

## LTs in the Development of Peri-Islet TLO

**Figure 1:**
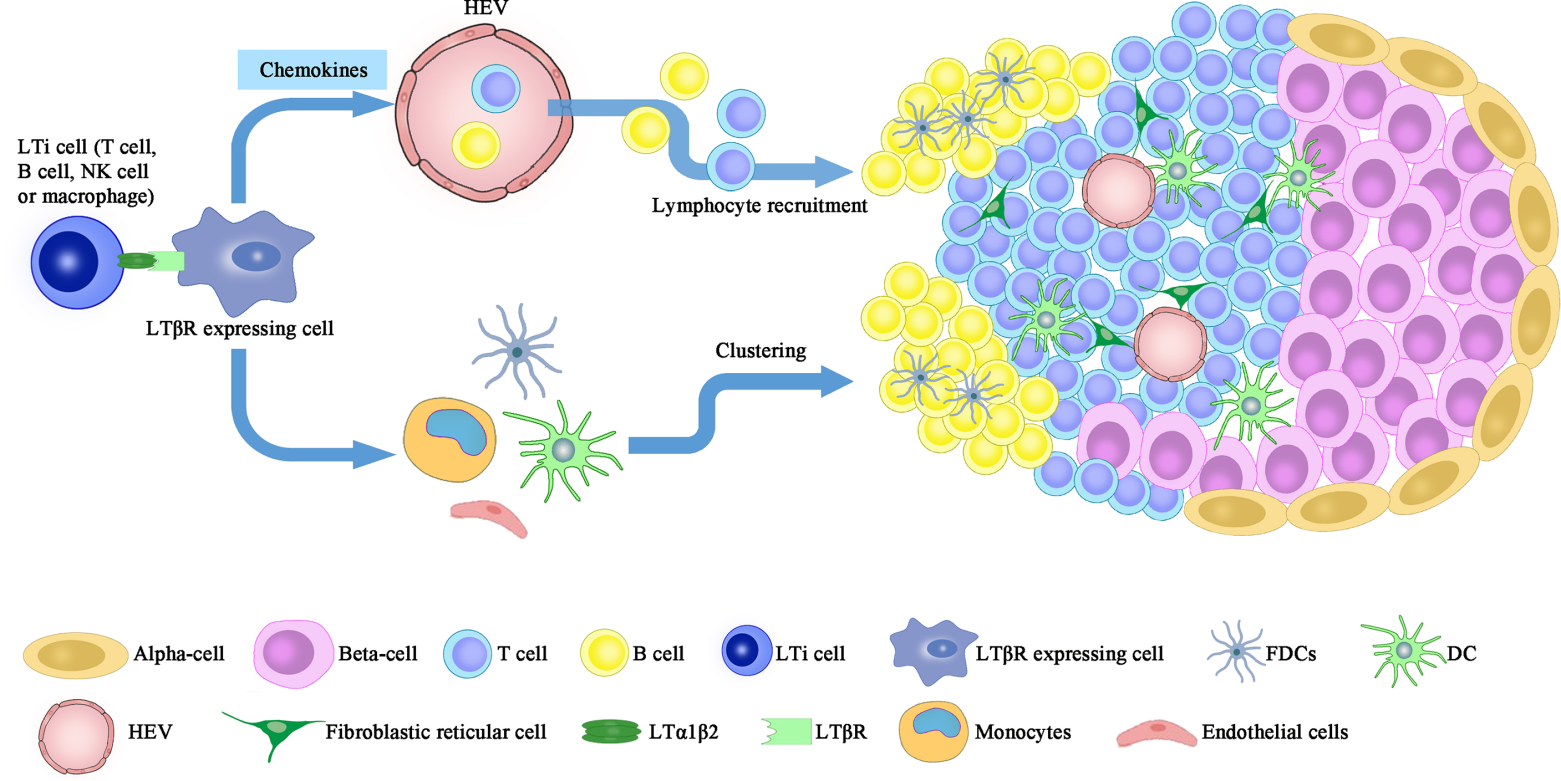
LT signaling participates in the formation of peri-islet TLO. Neogenesis of peri-islet TLO is orchestrated by the interaction between LTα1β2 expressing cells, including LTi, NK, B cells and activated T cells, and the LTβR expressing cells (FDCs, DCs, monocytes and stromal cells). This interaction induces islet infiltration of lymphocytes, which are then clustered into organized TLOs and contribute to β cell destruction. LTi, lymphoid tissue inducer; FDC, follicular dendritic cells; HEV, high endothelial vessels.

In the pancreas, artificial over-expression of chemokines including CCL19, CCL21 or CXCL13 in the pancreatic islets, together with the presence of IL-2 family cytokines, induce LTα1β2 expression on naïve T cells and mimic the neogenic process of TLO (39, 40). Ectopic development of TLO is a controlled multistep event that involves the recruitment of circulating immune cells (step 1), which is mediated by the CCL19/CCL21-CCR7 or CXCL13-CXCR5 axes; and the clustering of recruited cells (step 2), which relies partially on the IL-7Rα dependent LTα1β2 signals delivered by CD4^+^CD3^−^IL-7Rα^hi^ LTi cells (41) **(** **Figure 1** **)**.

Under the context of persistent insulitis, formation of TLO in the pancreas is a key immuno-pathologic feature (42). Chronic inflammatory lesions developed in the pancreas of RIP-LT mice (LT transgenic expression controlled by the rat insulin promoter) manifest intact cellular composition (e.g., T cells, B cells, plasma cells, and antigen-presenting cells), compartmentalized T and B cell regions, primary and secondary follicles, high endothelial venules (HEVs), and the ability to undergo immunoglobulin (Ig) class switch upon antigen challenge (43). Those observed characteristics support the formation of organized lymphoid tissue within the pancreas following sustained autoimmune insult. Moreover, LTβR is upregulated in aged NOD pancreas to promote the local formation of TLO once it’s ligated to TNFSF14 (LIGHT). In support of this notion, NOD mice with transgenic TNFSF14 expression in the islets develop spontaneous T1D even when pancreatic draining lymph nodes are absent (44).

It is worthy of note that TLOs could exist in a distinct immune state and largely differ between mice and humans. For example, unlike the transgenic TNFSF14 NOD mice, the Ins2-CCL21 transgenic NOD mice do not develop autoimmune diabetes due to the formation of “regulatory TLOs” around 4-week-old of age, which contain immunosuppressive Tregs (45). In sharp contrast, Tregs are essentially absent from human pancreatic TLOs and the formation of reticular fibers is not associated with CCL21. Particularly, studies in 21 donors exhibited TLOs diagnosed with clinical onset of T1D and insulitis, 12 of whom developed disease at younger age as compared to those lacking TLOs (46). Evidence also supports the formation of “inflammatory TLOs” under T1D setting, since administration of LTbetaR-Ig neutralizes the function of LTs, disassembles the established TLO and prevents T1D development at both early and late stage (47). Therefore, LT induced TLO remains a good interventional option for T1D treatment.

## LTs in Cytokine Induced β Cell Toxicity

**Figure 2:**
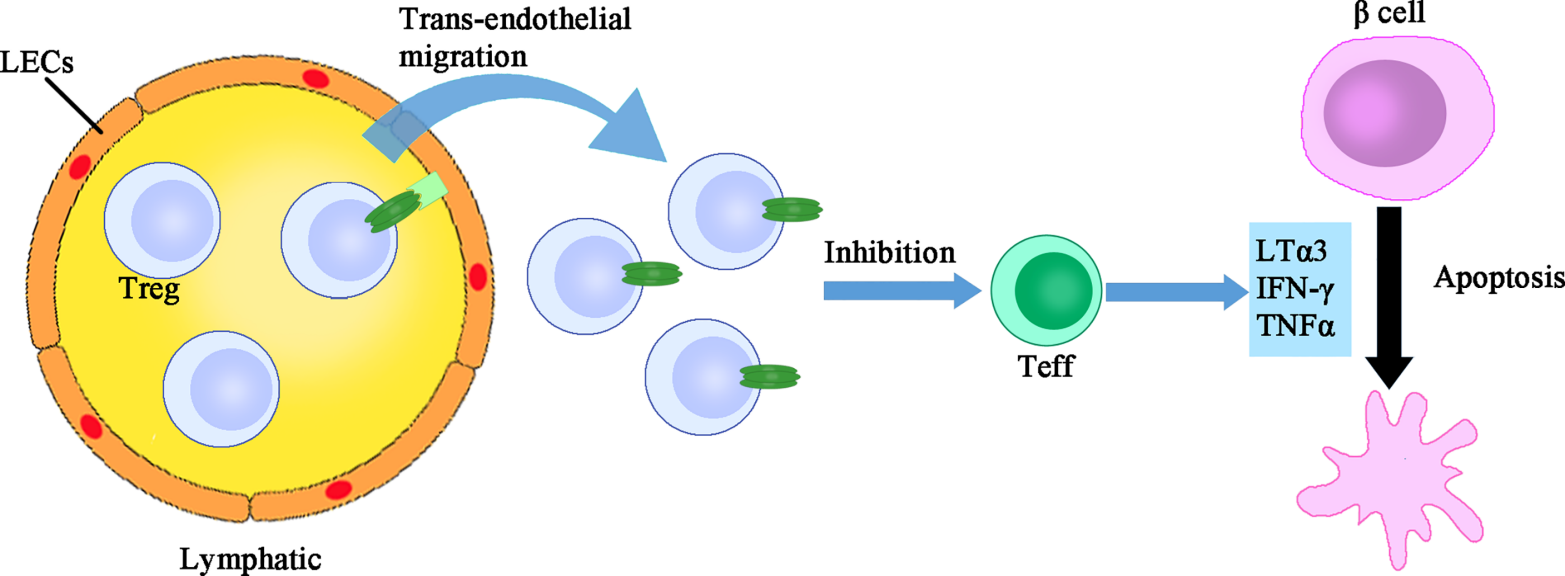
The direct immune regulatory function of LTs. LTs exert dual immune regulatory roles. One the one hand, LTα1β2 expressing Treg cells have more active trans-endothelial migration which facilitates their suppression of islet infiltrating Teff cells. On the other, LTα3 assists in the direct killing of β cells, although the toxicity of LTα3 alone is limited. LEC, lymphatic endothelial cells; Treg, regulatory T cells; Teff, effector T cells.

Once tested alone, the murine IFN-γ produces a mild dose-dependent lysis of islet cells, while human IFN-γ, murine IFN-α/β, IL-1, TNFα and LT have no effect. However, the combinations of TNFα (or LT) plus IL-1, TNFα (or LT) plus IFN-γ, and IL-1 plus IFN-γ exhibit synergistic cytotoxic effects, demonstrating a requirement of multiple cytokines for β cell destruction in T1D setting (48). As for glucose stimulated insulin secretion (GSIS), the hallmark of β cell functional capacity, it is suppressed by the treatment of TNFα or IFN-γ rather than by the stimulation of LT or IL-6, but synergistic effect on the suppression of GSIS between LT and IL-1β was observed (49). In another set of experiments, a half-maximal inhibition of GSIS after 7 days of culture was performed. The authors found that 100pg/ml of IL-1β is equivalent to 1000pg/ml of IL-1α in terms of inhibition of GSIS, while 25ng/ml of TNFα, 40ng/ml of LT or 25ng/ml of IFN-γ alone are not effective to impair GSIS or induce morphologic damage to islets (50). Taken together, although the toxicity of LTs in the pancreatic β cells is limited, they may, however, paly a subordinate role in the direct killing of β cells **(** **Figure 2** **)**.

## The Regulatory Role of LTs in Immune Responses

LTs are traditionally regarded as pro-inflammatory cytokines, but their role in the induction of inflammation is relatively weak as compared to that of TNFα. Indeed, pancreatic over-expression of TNFα resulted in overwhelming insulitis with majority of islets manifested infiltration. On the contrary, predominant peri-insulitis was observed in LTA transgenic mice, and LTA over-expression did not cause the reduction of insulin content nor led to the development of diabetes. These data suggest that LT alone is insufficient to initiate an inflammatory response strong enough to induce β cell destruction (51, 52). LTs may only possess a minor and ancillary effect on β cell toxicity (53). Distinct from most other cell types, islet β cells are not stimulated to express HLA class II by IFN-γ. However, IFN-γ in combination with either TNFα or LT induces HLA class II expression in β cells, which may enhance the antigen-presenting function of β cells and subsequent CD4 T cell activation (4, 54).

Surprisingly, LTs also display an unexpected anti-inflammatory function. Under the setting of islet allo-transplantation, circulating Treg cells firstly arrive in the allograft and then access into the draining lymph nodes *via* the afferent lymphatic vessel, thereby suppressing the overactive immune response. The lymphatic trans-endothelial migration process is essential for the suppressive function of Treg cells. Specifically, Treg surface LTα1β2 binds to LTβR expressed on the lymphatic endothelial cells (LECs), which then activates the non-canonical NF-κB signaling pathway and promotes the expression of adhesion molecules such as VCAM-1, thereby facilitating Treg trans-endothelial migration. Deficiency or blockade of LT abrogates Treg migration and accelerates islet allograft rejection (55). Furthermore, under the inflammatory condition of islet transplantation, activation of TLR2 on Treg cells augments LTα1β2-LTβR signaling and enhances the permissiveness of lymphatic vessel, thereby directing leukocytes out of tissue and promoting resolution of inflammation (56). Collectively, the immune regulatory function of LTs exhibits an opposing effect **(** **Figure 2** **)**. The pro-inflammatory or anti-inflammatory effect of LTs might hinge on the cell types affected, which warrants future investigations.

## Conclusion and Perspectives

LTs are multifunctional cytokines involved in T1D pathogenesis *via* promoting the formation of peri-islet TLOs, by which they synergize with other inflammatory mediators to enhance autoimmune responses and β cell destruction. It is worthy of note that LT alone manifests a relatively weak effect on β cell destruction. In this case, its contribution to T1D pathogenesis was almost neglected since the *in vivo* concentration of lymphotoxin is much lower than the dosage adopted in the *in vitro* studies. However, its impact on the formation of TLOs play a crucial role in autoimmune initiation and progression in T1D settings.

A critical feature for LTs is that they could exhibit both pro- and anti-inflammatory function. Previous studies demonstrated the role of LTs in stimulating immune responses. For instance, in LTA deficient mice, CD8 T cells fail to up-regulate activation or memory markers coupled with lower level of IL-12 receptor expression and impaired cytotoxic function (57). Blockade of membrane LT inhibits CD8 T cell mediated rejection of intestinal allograft, indicating that it could be a feasible target to prevent allograft rejection (58). Moreover, LTs not only regulate DC and CD4 T cell homeostasis at steady state, but are essential for maintaining the symbiotic relationship between the hosts and their microbiota (59). LTα1β2 expressed on Treg cells alters the permeability of lymphatic vessel and promotes the resolution of inflammatory response, which represents a novel aspect of Treg suppressive function. Nonetheless, as the relevant studies are focused on the islet transplantation model, whether such mechanism can be applied to Treg cells under the context of T1D settings and other autoimmune diseases is yet an open question.

Ectopic lymphoid neogenesis is a characteristic pathological event observed in tissues with chronic inflammation. During the process of insulitis, TLOs form around the islets and resemble the function of pancreatic draining lymph nodes. Administration of LTbetaR-Ig impedes TLO formation or disintegrates already formed TLOs, thereby alleviating the progression of T1D (47). However, B lymphocytes may not be significantly affected by TLO disassembly. Specifically, after blockade of chemoattractant CXCL13 to destroy the organization TLOs in the islets, B cells located in the “chaotic milieu” are comparable to those presented in the “organized TLOs” in terms of BCR diversity and the occurrence of somatic hypermutation (SHM) (60). Moreover, FTY-720 treatment aimed at maintaining TLO integrity also exhibited a protective effect on T1D, although the possibility of FTY-720 mediated blockade of the exit of lymphocytes from the pancreatic draining lymph nodes into the pancreatic tissue could not be excluded (61).

In summary, blockade of LT signaling could be a useful supplement in the early intervention of T1D. Given the complex nature of crosstalk between immune cells and β cells under T1D setting, blockade of LT signaling applied to the existing therapies could be an efficient approach to delay or even reverse the established T1D.

## Author Contributions

S-WL and FS wrote the manuscript; S-JR gave valuable suggestions and made critical revisions. TW and C-YW conceptualized and supervised the preparation of this manuscript. All authors contributed to the article and approved the submitted version.

## Funding

Our study was supported by the National Natural Science Foundation of China (82130023, 81920108009, 82100892, 82070808, 81873656, 82100823, 82100931, 91749207, 81770823 and 81800068), Department of Science and Technology of Hubei Province Program project (2020DCD014), the Postdoctoral Science Foundation of China (54000-0106540081 and 54000-0106540080), Hubei Health Committee Program (WJ2021ZH0002), the Integrated Innovative Team for Major Human Diseases Program of Tongji Medical College, Huazhong University of Science and Technology, and the Innovative Funding for Translational Research from Tongji Hospital.

## Conflict of Interest

The authors declare that the research was conducted in the absence of any commercial or financial relationships that could be construed as a potential conflict of interest.

## Publisher’s Note

All claims expressed in this article are solely those of the authors and do not necessarily represent those of their affiliated organizations, or those of the publisher, the editors and the reviewers. Any product that may be evaluated in this article, or claim that may be made by its manufacturer, is not guaranteed or endorsed by the publisher.

## Abbreviations

T1D, type 1 diabetes; LT, lymphotoxin; TNFα, tumor necrosis factor alpha; TLO, tertiary lymphoid organ; LTi, lymphoid tissue inducer; ILC, innate lymphoid cell; HVEM, herpes virus entry mediator; NOD, nonobese diabetic; HEV, high endothelial venule; GSIS, glucose stimulated insulin secretion; LEC, lymphatic endothelial cell; SHM, somatic hypermutation.
